# Prevalence and Associated Risk Factors for Hypovitaminosis D in Patients Scheduled for Primary Total Knee Arthroplasty in Germany

**DOI:** 10.3390/nu16233991

**Published:** 2024-11-21

**Authors:** Tizian Heinz, Miledi Hoxha, Philip Mark Anderson, Axel Jakuscheit, Manuel Weißenberger, Martin Lüdemann, Dominik Rak, Maximilian Rudert, Konstantin Horas

**Affiliations:** 1 Department of Orthopaedic Surgery, University of Wuerzburg, Koenig-Ludwig-Haus, Brettreichstr 11, 97074 Wuerzburg, Germany; miledihoxa@gmail.com (M.H.); axel.jakuscheit@klh.de (A.J.);; 2 Frankfurt Centre for Bone Health and Endocrinology, 60313 Frankfurt, Germany

**Keywords:** total knee arthroplasty, vitamin D insufficiency, osteoporosis, hypovitaminosis D, Germany, prevalence

## Abstract

Objective: Several studies have reported a high prevalence of hypovitaminosis D in orthopedic patients. The purpose of this prospective observational study was to report on the prevalence of hypovitaminosis D in patients scheduled for elective primary total knee arthroplasty (TKA) and its associated risk factors. Methods: In this monocentric cohort study, 25(OH) vitamin D serum levels were measured in 687 consecutive patients undergoing primary total knee arthroplasty (TKA) over a period of twelve months. Vitamin D levels were classified into deficiency (<20 ng/mL), insufficiency (20–29 ng/mL), and sufficiency (≥30 ng/mL). The study assessed the association of vitamin D levels with demographic and clinical factors, including age, sex, BMI, smoking status, and season of measurement. Statistical analyses included chi-square tests, correlation analyses, and multiple linear regression to identify significant predictors of vitamin D levels. Results: The cohort had a mean age of 67.70 ± 8.95 years and a mean BMI of 31.00 ± 5.71 kg/m^2^. Collectively, 33.9% of patients were vitamin D deficient, a further 32.9% were insufficient, and only 33.2% were sufficient. Vitamin D levels varied significantly with the season and were associated with obesity and smoking. Specifically, there was a small significant inverse correlation between BMI and vitamin D levels (r = −0.17, *p* < 0.01). Furthermore, regular nicotine abuse was linked to lower vitamin D levels (r = 0.14, *p* < 0.01). Multiple linear regression analysis reveals that age, BMI, nicotine abuse, and season were small significant predictors of preoperative vitamin D levels (R^2^ = 0.15, adjusted R^2^ = 0.12). A total of 121 (17.61%) patients reported routine vitamin D intake prior to surgery. Supplementing patients had a significantly higher mean serum vitamin D level and a significant reduction in the mean length of in-hospital stay (*p* < 0.01). Conclusions: The prevalence of vitamin D insufficiency and deficiency in patients undergoing elective primary TKA is alarmingly high. In ongoing efforts to optimize the efficacy and outcome of the TKA procedure, orthopedic surgeons should be advised to strongly implement the role of perioperative vitamin D levels in their routine practice.

## 1. Introduction

Recently, unprecedented interest in vitamin D serum levels in patients undergoing primary elective joint replacement surgery is evident in the scientific literature [1,2]. This may be due to the increasing understanding of the role and significance of vitamin D in maintaining a wide range of physiological functions. Vitamin D has been linked not only to bone metabolism through the regulation of calcium homeostasis and bone mineralization but also to muscle function and various autoimmune responses, affecting diseases such as diabetes, hypertension, multiple sclerosis, and cancer [3,4,5,6,7,8]. Vitamin D is obtained through nutritional sources (5–10%) and primarily through cutaneous synthesis under the influence of sunlight (80–90%). B photons from ultraviolet sunlight cause the photolysis of 7-dehydrocholesterol to precholecalciferol [9], which is then further processed to Vitamin D3 (cholecalciferol). Consequently, Vitamin D3 gets hydroxylated in the liver, resulting in the production of 25-hydroxyvitamin D (25[OH]D), known as the principal circulating form of Vitamin D. Following hydroxylation in the kidney, mediated by the enzyme 1α-hydroxylase, the biologically active dihydroxylated form, named calcitriol (1,25[OH]2D), is formed [7]. Calcitriol exerts its effects through specific vitamin D receptors, regulating not only calcium metabolism but also the differentiation and proliferation of various cell types. Notably, hypovitaminosis D is estimated to affect more than one billion people worldwide, rendering it the most common nutritional deficiency worldwide [7,10,11]. Meanwhile, the number of patients needing primary joint replacement surgery due to advanced osteoarthritis is estimated to increase sharply in the following years. Inacio et al. project an increase in the annual volume of primary total knee arthroplasty (TKA) of 69% by the year 2050 [12]. Similarly, Sloan et al. project the volume of annual TKA procedures to increase by even 85% [13]. These projections, paired with the knowledge of pandemic hypovitaminosis D, suggest a high rate of vitamin D insufficiency and deficiency among patients enrolled for primary TKA. Furthermore, some studies even suggest a link between hypovitaminosis D and the progression of osteoarthritis [14]. Of note, the prevalence of hypovitaminosis D depends largely on factors like geographic location, season, and vitamin D fortification of foods [9,15]. While various studies have explored the prevalence of hypovitaminosis D in a mostly broad and undefined orthopedic patient collective, data on the prevalence of vitamin D serum levels in patients undergoing primary TKA remain largely obscure [16,17]. At the same time, low serum vitamin D levels have been linked to increased hospitalization rates and worse outcomes following orthopedic surgical procedures [18,19,20]. Therefore, precise data on the prevalence and determinants of hypovitaminosis D in patients undergoing primary TKA are of utmost importance and have great potential to further improve patient safety and outcomes.

For this reason, it was the primary intention of this study to report on the prevalence and associated risk factors of hypovitaminosis D in a large monocentric patient cohort scheduled for primary total knee arthroplasty.

## 2. Materials and Methods

### 2.1. Study Design and Setting

This prospective study was approved by the local ethics committee (1/23-me), and written informed consent was obtained from all study participants. All patients were prospectively evaluated and included in the study. Patients were recruited from a single orthopedic university center located at 49.5 degrees north latitude. Patients were enrolled throughout the year.

### 2.2. Patient Cohort

All patients scheduled for primary total knee arthroplasty due to primary or secondary osteoarthritis of the knee were considered eligible for the study. The patient cohort consisted of 687 patients in total. The mean age was 67.70 ± 8.95 years, and the mean BMI was 31.00 ± 5.71 kg/m^2^. A total of 57.20% (393 patients) were female, and 42.80% (294 patients) were male. Enrollment commenced on 17 May 2023, concluding one year later. Limitations leading to exclusion from the study are defined as follows: (1) patients with an acute fracture around the knee undergoing arthroplasty, (2) any contraindication to subsequent vitamin D supplementation if low serum levels were detected, (3) lack of consent to participate in the study, and patients <18 years of age. If low serum vitamin D levels were detected, oral vitamin D supplementation was started the following day, usually the first postoperative day. Furthermore, patient demographics and characteristics (age, BMI, secondary diagnoses, etc.), as well as administrative data (length of stay, re-admission, etc.), were assessed.

### 2.3. Vitamin D Serum Level Measurements

Serum 25(OH) vitamin D levels as well as parathyroid hormone (PTH) and calcium levels were measured in eligible patients as part of the routine blood draw on admission (usually the day before surgery). Eligibility for the study was assessed during preoperative admission interviews. Serum 25(OH)D levels were assessed throughout the year, with sampling covering all seasons. The cobas^®^ 25-Hydroxyvitamin D Assay (Vitamin D Total) and the Elecsys PTH (1–84) Assay for the cobas^®^ e411 Analyzer (Roche Diagnostics, Mannheim, Germany) were used for all samples by the in-house laboratory.

Serum 25(OH)D levels were further classified according to the US Endocrine Society of Medicine [21]: (1) Deficiency: ≤19 ng/mL, (2) Insufficiency: 20 ng/mL to 29 ng/mL, and (3) Sufficiency: ≥30 ng/mL.

### 2.4. Surgical Procedure

A standard midline incision and parapatellar medial approach were used in every case. Regarding implants, a cruciate-retaining implant (NexGen CR Flex, Zimmer Biomet, Warsaw, IN, USA) was used in most cases. If the posterior cruciate ligament was not intact, a posterior stabilized (NexGen LPS, Zimmer Biomet, Warsaw, IN, USA) implant was used instead, and if a higher degree of instability was present, a semi-constrained model (LCCK, Zimmer Biomet, Warsaw, IN, USA) was used. If any known allergies against the implant (mostly nickel or cobalt) were known, a hypoallergenic implant was used (GMK Sphere SensiTin, Medacta International, Castel San Pietro, Switzerland). All implants were fixated using Palacos bone cement (Palacos R+G, Heraeus Medical, Wehrheim, Germany). Postoperatively, full weight-bearing as tolerated by pain was permitted, and early functional rehabilitation was commenced. A total of 2 g of tranexamic acid was instilled intraoperatively, followed by 500 mg intravenously 5–6 h after surgery as part of our institutional fast track concept (if no contraindication was present). This way, drains were widely avoided. Furthermore, intraoperative infiltration of the capsule and subcutaneous tissue was performed using local anesthetics, like ropivacaine, thereby lowering the postoperative demand for opioids.

### 2.5. Statistical Analysis

Statistical calculations were conducted using IBM SPSS statistical software (IBM Corp., 2022., IBM SPSS Statistics for Windows, Version 28.0., Armonk, NY, USA). For categorical data, absolute and relative frequencies were used. The normality of data distribution was assessed with Kolmogorov–Smirnov and Shapiro–Wilk tests. Depending on these assessments, either parametric or non-parametric tests were utilized accordingly. Differences in frequencies were evaluated using Chi-Square testing. The mean serum levels of vitamin D, PTH, and calcium were compared between groups using ANOVA and independent *t*-tests or their non-parametric equivalents when appropriate. Correlations were evaluated using linear and logistic regression analyses, including bivariate correlation tests. A *p*-value of less than 0.05 was considered statistically significant.

## 3. Results

### 3.1. Demographics

During the study period, the 25(OH) vitamin D serum levels of a total of 687 consecutive patients undergoing primary TKA and meeting the inclusion criteria were measured. Table 1 depicts the demographic and patient-specific characteristics of the study cohort. The proportions of male and female patients did not differ significantly. There was no statistically significant difference in age between female and male patients. However, female patients had a significantly higher mean BMI compared to males (*p* = 0.01) (Table 1). The proportion of smokers was not statistically different between the female and male groups (X^2^ = 3.22, df = 1, *p* = 0.07). The number of 25(OH) vitamin D measurements was equall throughout the year.

### 3.2. Vitamin D Status

**Figure 1:**
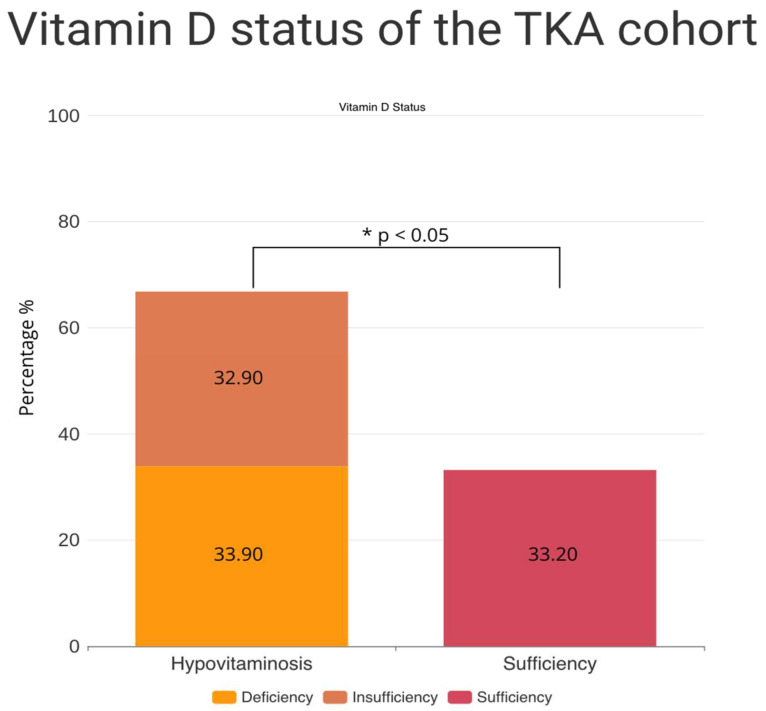
Vitamin D status of the TKA cohort. The prevalence of hypovitaminosis D (insufficiency and deficiency) was significantly higher than the proportion of patients with normal vitamin D serum levels. Significances are marked by asterisks (*).

**Figure 2:**
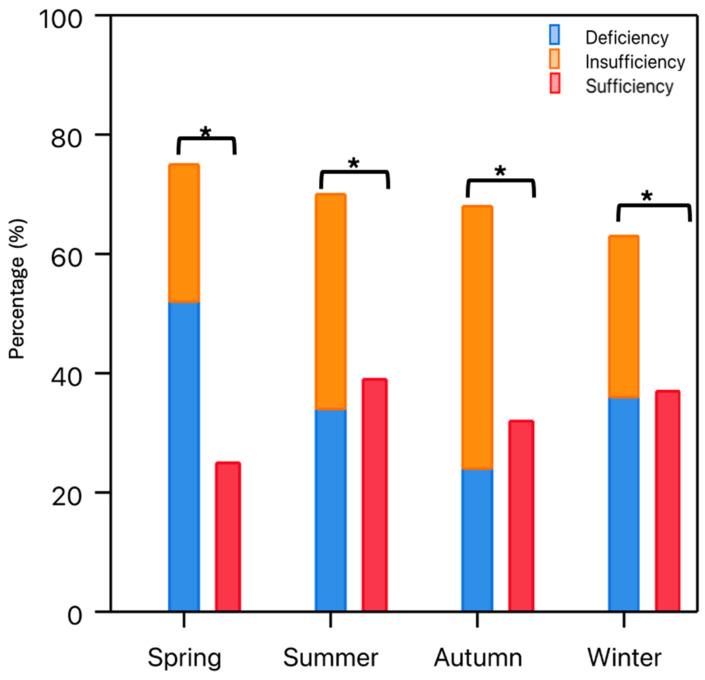
Bars indicate the relative frequency of patients with vitamin D deficiency, insufficiency, and deficiency depending on the season. The prevalence of hypovitaminosis D (insufficiency and deficiency) was significantly higher throughout the year compared to vitamin D sufficiency. Significances are marked by asterisks (*).

**Figure 3:**
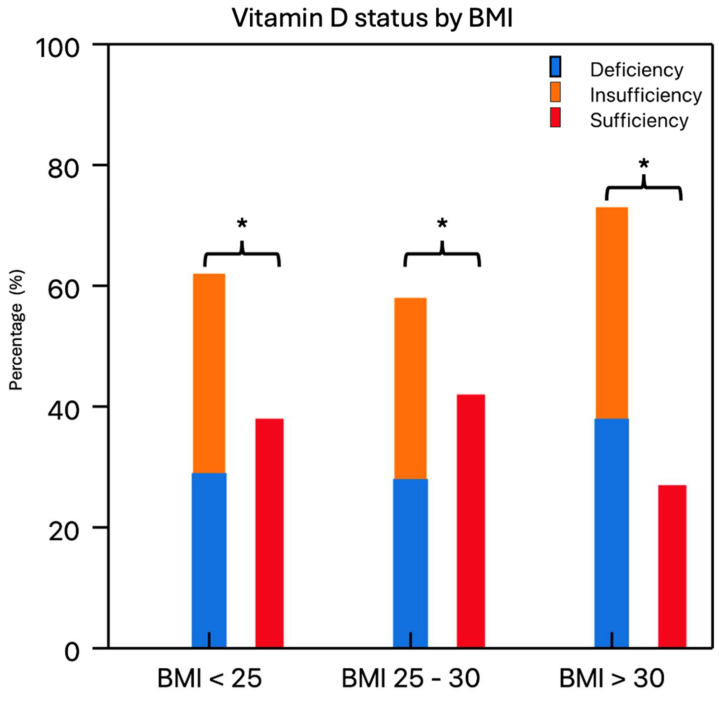
The relative frequency of hypovitaminosis D (vitamin D deficiency and sufficiency) was significantly higher in all BMI groups with a particularly high association of hypovitaminosis D in obese patients (BMI > 30 kg/m^2^). Significances are marked by asterisks (*).

The majority of patients undergoing primary TKA showed a vitamin D deficiency or vitamin D insufficiency (deficiency: 33.90%, insufficiency: 32.90%). The mean vitamin D level of the total cohort was 25.40 ± 11.70 ng/mL, and the vitamin D serum levels were not normally distributed throughout the cohort. There was a statistically significant difference in the prevalence of hypovitaminosis D (vitamin D deficiency and vitamin D insufficiency) and vitamin D sufficiency in the study cohort (hypovitaminosis: 66.81%, sufficiency: 33.19%, X^2^ = 77.67, df = 1, *p* < 0.01) (Figure 1). The prevalence of hypovitaminosis D was dependent on the season of sampling (X^2^ = 8.01, df = 3, *p* = 0.04), with a particularly high prevalence of hypovitaminosis D during spring (Figure 2). Furthermore, vitamin D status and obesity, as measured by the BMI, had a statistically significant association, with a remarkedly high proportion of hypovitaminosis D, especially among obese patients (BMI > 30 kg/m^2^) (X^2^ = 14.39, df = 2, *p* < 0.01) (Figure 3). Normal-weighted patients (BMI < 25 kg/m^2^) had a mean vitamin D serum level of 27.64 ng/mL compared to 23.71 ng/mL in obese (BMI > 30 kg/m^2^) patients (*p* = 0.01).

Among patients with regular nicotine abuse, the mean vitamin D serum levels were significantly lower compared to patients without regular nicotine abuse (non-smoker: 25.74 ng/mL ± 11.64, smoker: 21.07 ng/mL ± 11.34, *p* < 0.01).

Regarding the duration of in-hospital stay (length of stay, LOS), the mean residence time was 5.1 ± 2.04 days (min: 2, max: 22). The mean LOS was not significantly different between patients with hypovitaminosis D and those with adequate vitamin D levels (hypovitaminosis: 5.15 ± 2.09 days, sufficiency: 5.09 ± 1.96 days, *p* = 0.74).

A multiple linear regression analysis was performed to evaluate the effect of BMI, age, sex, nicotine abuse, the season of sampling, history of medication (glucocorticoids, L-thyroxine, proton-pump inhibitors, and aromatase inhibitors), and secondary diagnoses (diabetes, rheumatic disease, COPD, and carcinoma) on their potential influence as predictive variables for preoperative vitamin D serum levels. The multiple linear regression model explains a significant proportion of the variance of the preoperative vitamin D serum levels (R^2^ = 0.10, adjusted R^2^ = 0.07, *p* < 0.01). The regression coefficients are summarized in Table 2. BMI (*p* < 0.01), nicotine abuse (*p* < 0.01), rheumatic disease (*p* = 0.02), and a history of glucocorticoid or proton-pump inhibitor intake (*p* = 0.02) were significant predictors of preoperative vitamin D serum levels.

Out of the 687 patients enrolled in this study, a total of 121 (17.61%) patients reported regular vitamin D intake prior to surgery. Among the patients with regular vitamin D supplementation, only 32 individuals (26.45%) had hypovitaminosis D. Patients with regular vitamin D intake showed significantly higher mean vitamin D serum levels compared to individuals without intake prior to surgery (35.55 ng/mL vs. 23.05 ng/mL, *p* < 0.01). Furthermore, among patients with regular supplementation, a statistically significant decrease in the mean LOS was observable (Table 3). Furthermore, patients who reported a regular vitamin D intake were significantly older than patients without supplementation (Table 3). Patients who supplemented vitamin D on a regular basis yielded significantly higher serum vitamin D levels throughout the year.

## 4. Discussion

The main finding of this study is an alarmingly high prevalence of hypovitaminosis D in patients undergoing routine primary TKA. With a number as high as 68% of patients in this specific study cohort presenting with unacceptably low serum vitamin D levels prior to primary TKA, the potential scope and impact on the postoperative trajectory may be of high clinical significance. Further, we identified smoking and a high BMI as potential risk factors for low vitamin D levels. Moreover, rheumatic disease and a history of a specific medication intake (glucocorticoids or proton-pump inhibitors) seem to impact preoperative vitamin D status.

This reported prevalence is in line with data well-known from the literature. Emara et al. conducted a systematic review on the prevalence of hypovitaminosis D in patients undergoing TKA and THA and found a prevalence of 53.4% for vitamin D insufficiency and 39.4% for vitamin D deficiency, which resembles the values reported in our study [22]. In a survey including approximately 7000 patients, Rabenberg et al. found an even higher prevalence of 61.6% for vitamin D deficiency in the general German population aged 18 to 79 years [23]. In another nationwide study including more than 1200 patients with permanent residency in Germany, the prevalence of vitamin D insufficiency and deficiency was found to be 17% and 75%, respectively [24]. However, the direct comparison of hypovitaminosis D prevalence in the literature is often hampered by the usage of inconsistent thresholds for defining vitamin D insufficiency and deficiency. In the present study, thresholds commonly reported in the field of orthopedics and recommended by The Endocrine Society were used, thus easing comparability with other scientific literature on the same research topic [10,21,25,26]. While the general prevalence of hypovitaminosis D is considered to be high in the general population, various studies have demonstrated that especially orthopedic patient clientele is prone to particularly high rates of vitamin D insufficiency and deficiency, reaching values of up to 97% with inadequate serum 25(OH) vitamin D levels [17,27,28]. However, while most of the studies have reported on the prevalence of hypovitaminosis D in an unselected orthopedic patient cohort, including patients scheduled both for conservative surgical treatment, information on the prevalence and potential implications of the vitamin D status in a patient cohort specifically enrolled for TKA due to advanced OA of the knee remains largely obscure [29,30]. To the knowledge of the authors, this is the first study reporting on hypovitaminosis D prevalence in a large patient cohort scheduled for primary TKA in Germany.

While the intra- and extraosseous functions of vitamin D are well known, and severely decreased vitamin D levels are associated with various diseases, like rachitis, inadequate vitamin D concentrations have been linked with several unfavorable effects following TKA [31,32]. Moreover, it has been demonstrated that patients with decreased articular cartilage thickness are more likely to be insufficient in vitamin D; thus, a low vitamin D status might be a risk factor for the development of osteoarthritis [33]. Nonetheless, other studies have failed to report a clear association between vitamin D deficiency and osteoarthritis. Hence, further studies are required to elucidate the role of vitamin D in osteoarthritis [34]. In addition, vitamin D is important for good muscle function, the osseointegration of orthopedic implants, and the outcome of patients undergoing joint arthroplasty [34]. Hedge et al. linked vitamin D deficiency prior to planned TKA procedures to a significantly increased risk of periprosthetic infections and a higher rate of explanted prosthesis one year following the index procedure [35]. Furthermore, low vitamin D concentrations in TKA patients have also been associated with impaired postoperative functional outcomes, a higher rate of postoperative stiffness, and even non-implanted related complications, like myocardial infections and deep vein thrombosis, have been shown to be remarkedly higher in patients with low preoperative vitamin D serum levels [35,36]. These observations are in line with a reported inverse correlation between preoperative vitamin D levels and the mean duration of in-hospital stays [1,36]. Multiplying the potential adverse implications of hypovitaminosis D on the subsequent TKA procedure with the reported high prevalence of vitamin D insufficiency and deficiency in arthroplasty patients raises the question of whether there is an indication for universally preoperative screening and vitamin D repletion. From an economic point of view, studies have claimed tremendous cost savings both by selective and non-selective vitamin D repletion prior to TKA due to the projected reduction in potential complications [37]. There is also supporting evidence that timely preoperative vitamin D supplementation can correct most serum vitamin D levels within several weeks [38]. The data from this study cohort have furthermore impressively demonstrated how a regular vitamin D intake can successfully prevent inadequate serum vitamin D levels. Moreover, our data suggest that patients with regular vitamin D supplementation demonstrate enhanced recovery by a significant reduction in the mean LOS.

Another major finding of the present study was the identification of the risk factors associated with hypovitaminosis D in elective TKA patients. Thereby, it could be demonstrated that obesity was a significant and independent risk factor for preoperatively inadequate vitamin D levels. This is in line with previous findings reporting an association between vitamin D deficiency and obesity [17,39]. A possible explanation for this observation is that vitamin D is tightly bound in fatty tissues, and as such, less circulating vitamin D is available [39]. Furthermore, preoperative nicotine abuse increased the risk for hypovitaminosis D significantly. Moreover, the season of vitamin D sampling was an independent risk factor for inadequate preoperative vitamin D levels, with particularly low values in spring and winter. Vitamin D deficiency is generally known to decrease during months with a higher amount of daily sun exposure [40].

## 5. Conclusions

The prevalence of hypovitaminosis D in patients undergoing elective primary TKA is alarmingly high. In ongoing efforts to optimize the efficacy and outcome of the TKA procedure, orthopedic surgeons should be advised to strongly implement the role of perioperative vitamin D levels in their routine practice.

## Figures and Tables

**Table 1:** Patient characteristics.

Patient Characteristics	Value
Sex	
Male (number, percent)	294, 42.8%
Female (number, percent)	393, 57.2%
Age (years ± SD)	67.70 ± 8.95
Male	61.10 ± 8.59
Female	68.20 ± 9.20
BMI (kg/m^2^, ± SD)	31.00 ± 5.71
Male	30.33 ± 5.34
Female	31.59 ± 5.94
Smoker (number, percent)	57, 8.30%
Comorbidities (number, percent)	
Diabetes	71, 10.30%
Rheumatoid arthritis	20, 2.90%
Tumor disease	7, 1.00%
Chronic obstructive pulmonary disease (COPD)	8, 1.20%
Chronic kidney disease	
Stage II	384, 55.90%
Stage III	75, 10.90%
Stage IV	3, 0.40%

**Table 2:** Predictors of preoperative vitamin D serum levels.

Predictor	Coefficient	Std. Error	T-Statistic	*p*-Value
Sex	1.30	0.95	1.37	0.17
Age	−0.03	0.0	−0.54	0.59
BMI	−0.43	0.08	−5.15	<0.01
Nicotine abuse	−4.97	1.66	−2.99	<0.01
LOS	−0.16	0.24	−0.66	0.51
Secondary diagnoses (rheumatic disease)	6.46	2.69	2.41	0.02
History of medication (glucocorticoids or proton-pump inhibitors)	1.36	0.89	1.53	0.02
Season	−0.06	1.01	−0.06	0.95

**Table 3:** Impact of vitamin D supplementation on LOS.

	Regular Vit D Intake	No Regular Vit D Intake	*p*-Value
Mean LOS (±SD)	5.02 ± 2.00	5.63 ± 2.08	<0.01
Age	69.51 ± 8.71	67.33 ± 8.97	0.02
BMI	31.00 ± 6.21	30.99 ± 5.60	0.98
Sex (m/f)	m: 26.45% f: 73.55%	m: 46.29% f: 53.71%	<0.01

## Data Availability

The original contributions presented in the study are included in the article, further inquiries can be directed to the corresponding author.

## References

[1] Vivek K., Kamal R., Perera E., Gupte C.M. (2024). Vitamin D Deficiency Leads to Poorer Health Outcomes and Greater Length of Stay After Total Knee Arthroplasty and Supplementation Improves Outcomes. JBJS Rev..

[2] Birinci M., Hakyemez Ö.S., Geçkalan M.A., Mutlu M., Yildiz F., Bilgen Ö.F., Azboy İ. (2024). Effect of Vitamin D Deficiency on Periprosthetic Joint Infection and Complications After Primary Total Joint Arthroplasty. J. Arthroplast..

[3] Garland C.F., Comstock G.W., Garland F.C., Helsing K.J., Shaw E.K., Gorham E.D. (1989). Serum 25-hydroxyvitamin D and colon cancer: Eight-year prospective study. Lancet.

[4] Garland F.C., Garland C.F., Gorham E.D., Young J.F. (1990). Geographic variation in breast cancer mortality in the United States: A hypothesis involving exposure to solar radiation. Prev. Med..

[5] Pittas A.G., Harris S.S., Stark P.C., Dawson-Hughes B. (2007). The effects of calcium and vitamin D supplementation on blood glucose and markers of inflammation in nondiabetic adults. Diabetes Care.

[6] Hernán M.A., Olek M.J., Ascherio A. (1999). Geographic variation of MS incidence in two prospective studies of US women. Neurology.

[7] Mithal A., Wahl D.A., Bonjour J.P., Burckhardt P., Dawson-Hughes B., Eisman J.A., El-Hajj Fuleihan G., Josse R.G., Lips P., Morales-Torres J. (2009). Global vitamin D status determinants of hypovitaminosis, D. Osteoporos. Int..

[8] Rostand S.G. (1997). Ultraviolet light may contribute to geographic and racial blood pressure differences. Hypertension.

[9] Holick M.F. (1995). Environmental factors that influence the cutaneous production of vitamin D. Am. J. Clin. Nutr..

[10] Holick M.F. (2007). Vitamin D Deficiency. N. Engl. J. Med..

[11] Maier G.S., Horas K., Seeger J.B., Roth K.E., Kurth A.A., Maus U. (2015). Vitamin D insufficiency in the elderly orthopaedic patient: An epidemic phenomenon. Int. Orthop..

[12] Inacio M.C.S., Paxton E.W., Graves S.E., Namba R.S., Nemes S. (2017). Projected increase in total knee arthroplasty in the United States—An alternative projection model. Osteoarthr. Cartil..

[13] Sloan M., Premkumar A., Sheth N.P. (2018). Projected Volume of Primary Total Joint Arthroplasty in the U.S., 2014 to 2030. J. Bone Jt. Surg..

[14] Chevalley T., Brandi M.L., Cashman K.D., Cavalier E., Harvey N.C., Maggi S., Cooper C., Al-Daghri N., Bock O., Bruyère O. (2022). Role of vitamin D supplementation in the management of musculoskeletal diseases: Update from an European Society of Clinical and Economical Aspects of Osteoporosis, Osteoarthritis and Musculoskeletal Diseases (ESCEO) working group. Aging Clin. Exp. Res..

[15] Gerdhem P., Ringsberg K.A.M., Obrant K.J., Akesson K. (2005). Association between 25-hydroxy vitamin D levels, physical activity, muscle strength and fractures in the prospective population-based OPRA Study of Elderly Women. Osteoporos. Int..

[16] Piuzzi N.S., George J., Khlopas A., Klika A.K., Mont M.A., Muschler G.F., Higuera C.A. (2018). High prevalence and seasonal variation of hypovitaminosis D in patients scheduled for lower extremity total joint arthroplasty. Ann. Transl. Med..

[17] Maier G.S., Seeger J.B., Horas K., Roth K.E., Kurth A.A., Maus U. (2015). The prevalence of vitamin D deficiency in patients with vertebral fragility fractures. Bone Jt. J..

[18] Shin K.-Y., Park K.K., Moon S.-H., Yang I.H., Choi H.-J., Lee W.-S. (2017). Vitamin D deficiency adversely affects early post-operative functional outcomes after total knee arthroplasty. Knee Surg. Sports Traumatol. Arthrosc..

[19] Jansen J.A., Haddad F.S. (2013). High prevalence of vitamin D deficiency in elderly patients with advanced osteoarthritis scheduled for total knee replacement associated with poorer preoperative functional state. Ann. R. Coll. Surg. Engl..

[20] Maniar R.N., Patil A.M., Maniar A.R., Gangaraju B., Singh J. (2016). Effect of Preoperative Vitamin D Levels on Functional Performance after Total Knee Arthroplasty. Clin. Orthop. Surg..

[21] Holick M.F., Binkley N.C., Bischoff-Ferrari H.A., Gordon C.M., Hanley D.A., Heaney R., Hassan Murad M., Weaver C.M. (2011). Evaluation, Treatment, and Prevention of Vitamin D Deficiency: An Endocrine Society Clinical Practice Guideline. J. Clin. Endocrinol. Metab..

[22] Emara A.K., Nageeb E., George J., Buttaro M.A., Higuera C., Piuzzi N.S. (2020). Hypovitaminosis D in lower extremity Joint Arthroplasty: A systematic review and meta-analysis. J. Orthop..

[23] Rabenberg M., Scheidt-Nave C., Busch M.A., Rieckmann N., Hintzpeter B., Mensink G.B. (2015). Vitamin D status among adults in Germany—Results from the German Health Interview and Examination Survey for Adults (DEGS1). BMC Public Health.

[24] Zittermann A., von Helden R., Grant W., Kipshoven C., Ringe J.D. (2009). An estimate of the survival benefit of improving vitamin D status in the adult german population. Derm. Endocrinol..

[25] Jeon Y.-D., Cho S.-D., Youm Y.-S., Song J.-Y., Lee K.-J., Park K.-B. (2022). The Prevalence of Vitamin D Deficiency in Patients Undergoing Total Knee Arthroplasty: A Propensity Score Matching Analysis. Arch. Osteoporos..

[26] Maier G.S., Maus U., Lazovic D., Horas K., Roth K.E., Kurth A.A. (2016). Is there an association between low serum 25-OH-D levels and the length of hospital stay in orthopaedic patients after arthroplasty?. J. Orthop. Traumatol..

[27] Andres B.A., Childs B.R., Vallier H.A. (2018). Treatment of Hypovitaminosis D in an Orthopaedic Trauma Population. J. Orthop. Trauma.

[28] Goula T., Kouskoukis A., Drosos G., Tselepis A.-S., Ververidis A., Valkanis C., Zisimopoulos A., Kazakos K. (2015). Vitamin D status in patients with knee or hip osteoarthritis in a Mediterranean country. J. Orthop. Traumatol..

[29] Santhanagopal S., Sebastian M., Muniswamy M.M., Pilar A. (2020). A study on vitamin D status among orthopaedic patients. Int. J. Res. Orthop..

[30] Maier G.S., Jakob P., Horas K., Roth K.E., Kurth A.H.A., Maus U. (2013). Vitamin D deficiency in orthopaedic patients: A single center analysis. Acta Orthop. Belg..

[31] Doppelt S.H. (1984). Vitamin D, rickets, and osteomalacia. Orthop. Clin. N. Am..

[32] Biasucci G., Donini V., Cannalire G. (2024). Rickets Types and Treatment with Vitamin D and Analogues. Nutrients.

[33] Garfinkel R.J., Dilisio M.F., Agrawal D.K. (2017). Vitamin D and Its Effects on Articular Cartilage and Osteoarthritis. Orthop. J. Sports Med..

[34] Maier G.S., Weissenberger M., Rudert M., Roth K.E., Horas K. (2021). The role of vitamin D and vitamin D deficiency in orthopaedics and traumatology-a narrative overview of the literature. Ann. Transl. Med..

[35] Hegde V., Arshi A., Wang C., Buser Z., Wang J.C., Jensen A.R., Adams J.S., Zeegen E.N., Bernthal N.M. (2018). Preoperative Vitamin D Deficiency Is Associated with Higher Postoperative Complication Rates in Total Knee Arthroplasty. Orthopedics.

[36] Jansen J.A., Tahmassebi J., Haddad F.S. (2018). Vitamin D Deficiency Is Associated with Longer Hospital Stay and Lower Functional outcome After Total Knee Arthroplasty. Acta Orthop. Belg..

[37] Arshi A., Shieh A., Adams J.S., Bernthal N.M., Zeegen E.N., Sassoon A.A. (2019). Preoperative Vitamin D Repletion in Total Knee Arthroplasty: A Cost-Effectiveness Model. J. Arthroplast..

[38] Mouli V.H., Schudrowitz N., Carrera C.X., Uzosike A., Fitz W., Rajaee S.S. (2021). High Dose Vitamin D Supplementation Can Correct Hypovitaminosis D Prior to Total Knee Arthroplasty. J. Arthroplast..

[39] Alzohily B., AlMenhali A., Gariballa S., Munawar N., Yasin J., Shah I. (2024). Unraveling the complex interplay between obesity and vitamin D metabolism. Sci. Rep..

[40] Klingberg E., Oleröd G., Konar J., Petzold M., Hammarsten O. (2015). Seasonal variations in serum 25-hydroxy vitamin D levels in a Swedish cohort. Endocrine.

